# Dopamine receptor agonists modulate voluntary alcohol intake independently of individual levels of alcohol intake in rats

**DOI:** 10.1007/s00213-016-4330-x

**Published:** 2016-05-28

**Authors:** Marcia Spoelder, Annemarie M. Baars, Marthe D. Rotte, Louk J. M. J. Vanderschuren, Heidi M. B. Lesscher

**Affiliations:** Department of Animals in Science and Society, Division of Behavioural Neuroscience, Faculty of Veterinary Medicine, Utrecht University, Yalelaan 2, 3584 CM Utrecht, The Netherlands

**Keywords:** Addiction, Alcohol, Dopamine receptor, Individual differences

## Abstract

**Rationale:**

Individual susceptibility to alcohol use disorder has been related to functional changes in dopaminergic neurotransmission.

**Objectives:**

The aim of the current work was to assess the effects of selective dopamine D1 and D2 receptor agonists and antagonists on alcohol consumption in rats that differ in individual levels of alcohol intake.

**Methods:**

The effects of the dopamine D1 receptor agonist SKF 82958, the dopamine D1 receptor antagonist SCH 23390, the dopamine D2 receptor agonist sumanirole and the dopamine D2 receptor antagonist L741,626 on alcohol consumption and preference were assessed at different time points after treatment in subgroups of low and high alcohol drinking rats (LD and HD) using an intermittent alcohol access paradigm.

**Results:**

SKF 82958 decreased alcohol intake and alcohol preference throughout the 24-h session. Sumanirole decreased alcohol intake during the first 2 h, but increased alcohol intake during the remainder of the session. The effects of SKF 82958 and sumanirole on alcohol intake and alcohol preference were comparable in LD and HD. By contrast, the dopamine receptor antagonists SCH 23390 and L741,626 did not alter alcohol consumption in either group at any time point.

**Conclusions:**

These data indicate that stimulation of dopamine D1 receptors reduces alcohol intake, but that endogenous dopamine does not play a primary role in alcohol consumption. Moreover, the difference in alcohol consumption between LD and HD does not involve altered dopamine signaling.

## Introduction

Alcohol use disorder (AUD) is a chronic relapsing brain disorder, which is characterized by compulsive engagement in alcohol use (American Psychiatric Association 2013). There is substantial heterogeneity in both the etiology and expression of AUD. Several (e.g. genetic, environmental and personality) factors are thought to contribute to the individual vulnerability for this disorder (Chassin et al. 2002; Anderson 2006; Perry and Carroll 2008; Goudriaan et al. 2011; Enoch 2013). More insight into the mechanisms underlying individual variation in alcohol consumption may provide important knowledge about the development of AUD, which may contribute to improved personalized treatments for AUD.

One prominent hypothesis is that variations in dopaminergic neurotransmission underlie the individual susceptibility to AUD (Noble 2000; Tupala and Tiihonen 2004; Le Foll et al. 2009). The mesolimbic dopamine system has been widely implicated in motivated-, including alcohol-directed behaviour (Berridge 2007; Robbins and Everitt 2007; Spanagel 2009; Volkow et al. 2011; Salamone and Correa 2012; Floresco 2015; Korpi et al. 2015). Acute alcohol administration has been shown to activate dopamine neuron firing in the ventral tegmental area (VTA) (Gessa et al. 1985; Brodie et al. 1990; Brodie et al. 1999), and alcohol ingestion increases dopamine release in the ventral striatum (Weiss et al. 1993; Boileau et al. 2003; Doyon et al. 2003). Moreover, acute and repeated alcohol exposure has been shown to alter dopaminergic function at both the pre- and postsynaptic level (Reggiani et al. 1980; Imperato et al. 1987; Imperato and Di Chiara 1988; Nestby et al. 1997; Nestby et al. 1999; Gonzales et al. 2004; Sari et al. 2006).

The actions of dopamine are mediated by two principal classes of dopamine receptor subtypes, i.e. the D1-like (D1/D5) and D2-like (D2/D3/D4) dopamine receptors (Le Foll et al. 2009). However, the relative contributions of the different dopamine receptor subtypes to the development and maintenance of AUD remain incompletely understood. In addition, it is unknown whether individual susceptibility to AUD relates to a specific dopamine receptor subtype. Alterations in dopamine D2 receptor function have been the main focus in AUD studies over the last decade (Noble 2000; Connor et al. 2002; Kraschewski et al. 2009). Thus, reduced levels of dopamine D2 receptors in limbic areas have been observed in both AUD patients (Hietala et al. 1994; Volkow et al. 1996; Tupala et al. 2001; Volkow et al. 2002; Tupala et al. 2003) and in alcohol-preferring rats and mice (Stefanini et al. 1992; McBride et al. 1993; Zhou et al. 1995; Bice et al. 2008). The dopamine D1 receptor has also been implicated in alcohol seeking and consumption. Both dopamine D1 and D2 receptor deficient mice show marked reductions in alcohol-directed behaviour (El-Ghundi et al. 1998; Phillips et al. 1998; Risinger et al. 2000; Thanos et al. 2005). Moreover, involvement of both dopamine receptor subtypes in alcohol consumption and reinforcement has been demonstrated (Linseman 1990; Silvestre et al. 1996; Files et al. 1998; Cohen et al. 1999; Melendez et al. 2005; Ding et al. 2015).

The aim of this study was to determine the contribution of dopamine D1 and D2 receptors to individual differences in alcohol consumption under intermittent alcohol access (IAA) conditions. IAA results in high and escalating levels of alcohol intake, indicating that this paradigm is well suited to investigate biological mechanisms of AUD (Wise 1973; Simms et al. 2008; Hopf et al. 2010; Lesscher et al. 2010; Loi et al. 2010; Hwa et al. 2011; Sabino et al. 2013; Spoelder et al. 2015). We recently observed marked individual differences in alcohol intake in outbred rats using the IAA paradigm, which was related to the motivational properties of alcohol and measures of compulsive alcohol intake (Spoelder et al. 2015). We therefore used the IAA paradigm to determine the effects of dopamine D1 and D2 receptor-selective agonists and antagonists on voluntary alcohol consumption in groups of high (HD) and low alcohol drinking (LD) rats. We hypothesized that, if variations in dopamine neurotransmission underlie individual vulnerability to AUD, treatment with dopaminergic compounds should have differential effects on alcohol intake in HD and LD.

## Materials and methods

### Animals

Male Lister Hooded rats (Charles River, Germany) weighing 320–360 g at the start of the experiment were used. The rats were housed individually under controlled temperature and humidity conditions, a reversed light/dark cycle (lights off 7.00 AM), with ad libitum access to water and chow at all times. All rats were weighed and handled at least once per week throughout the experiment. All experiments were approved by the Animal Ethics Committee of Utrecht University and conducted in agreement with Dutch laws (Wet op de dierproeven, 1996) and European regulations (Guideline 86/609/EEC).

### Intermittent alcohol access in the home-cage

The rats were provided access to 20 % alcohol (*v*/*v*) and water in a two-bottle choice IAA setup in the home-cage for 3 days a week (Monday-Wednesday-Friday) using bottles that were fitted with stainless-steel dual ball bearing drinking spouts. Bottle positions were switched between sessions to avoid side bias. Rats were provided with access to alcohol for 7 h/day in the first month. Subsequently, access to alcohol was extended to 24 h/day in the second month and for the remainder of the experiment. The bottles were weighed prior to and after each session to calculate alcohol intake (g/kg) and alcohol preference (% of total fluid consumed). The selection of LD and HD was performed as previously described (Spoelder et al. 2015). Briefly, after 2 months of IAA, the rats were ranked based on the animals’ average alcohol intake per week and were assigned ranking scores. The weekly ranking scores were summed to calculate a total ranking score per rat. The rats within the lower and upper 25 % of the total ranking score range were designated as LD and HD, respectively. The middle 50 %, designated as medium alcohol drinking rats, were used in other experiments.

### Drugs

Alcohol (99.5 %, Klinipath, The Netherlands) was freshly diluted with tap water once per week to 20 % (*v*/*v*). The dopamine D1 receptor agonist SKF 82958 hydrobromide ((±)-6-Chloro-7,8-dihydroxy-3-allyl-1-phenyl-2,3,4,5-tetrahydro-1H-3-benzazepine hydrobromide) and the dopamine D2 receptor agonist sumanirole maleate ((R)-5,6-Dihydro-5-(methylamino)-4H-imidazo[4,5,1-ij]quinolin-2(1H)-one maleate) were generously supplied by the NIMH Chemical Synthesis and Drug Supply Program, Bethesda, MD, USA. The dopamine D1 receptor antagonist SCH 23390 hydrochloride (R(+)-7-Chloro-8-hydroxy-3-methyl-1-phenyl-2,3,4,5-tetrahydro-1H-3-benzazepine hydrochloride) and the dopamine D2 receptor antagonist L741,626 ((±)-3-[4-(4-Chlorophenyl)-4-hydroxypiperidin-l-yl]methyl-1H-indole) were purchased from Tocris (UK). SKF 82958, sumanirole and SCH 23390 were dissolved in sterile saline (0.9 % NaCl). L741,626 was dissolved in 5 % polyethylene glycol (PEG) and 5 % Tween 80 in Milli-Q water. Saline was used as a vehicle for SKF 82958, sumanirole and SCH 23390; a 5 % PEG/Tween solution served as the vehicle for L741,626 treatments. Drug solutions were freshly prepared daily.

### Drug administration and injection procedures

All drug solutions were administered subcutaneously in a volume of 1 ml/kg body weight, 20 min prior to the drinking session in the home cage according to a within-subject Latin square design. Alcohol and water bottles were weighed before each session and 2, 7 and 24 h after the start of the session. Because the effects of the drugs were examined under IAA, each treatment session was always followed by at least one alcohol-free day that also served as washout day. Thereafter, there was at least one drug-free re-baseline session between sessions for the same drug and there were at least three re-baseline sessions between different drugs. Two batches of rats were used for this study; the rats in the first batch were treated with the dopamine D2 receptor agonist sumanirole (0, 0.1, 0.3 and 1.0 mg/kg) and the dopamine D2 receptor antagonist L741,626 (0, 0.3, 1.0, and 3.0 mg/kg) in a counterbalanced fashion. The rats in the second batch were treated with the dopamine D1 receptor agonist SKF 82958 (0, 0.3, 1.0 and 3.0 mg/kg) and the dopamine D1 receptor antagonist SCH 23390 (0, 3, 10 and 30 μg/kg). In addition, the effects of the highest dose of sumanirole (0 and 1.0 mg/kg) and L741,626 (0 and 3.0 mg/kg) on alcohol consumption were replicated in this second batch. The order of drugs administered in the second batch was similar for each animal; the rats were first treated with SCH 23390, followed by sumanirole, SKF 82958 and L741,626. All rats received two habituation injections (1.0 ml/kg saline (0.9 % NaCl) subcutaneously), prior to alcohol drinking sessions 1 week before actual drug testing began. The doses of the dopamine receptor agonists and antagonists are based on previous studies that report behavioural effects of these compounds within these dose ranges (Linseman 1990; Dyr et al. 1993; George et al. 1995; Gnanalingham et al. 1995; Silvestre et al. 1996; El-Ghundi et al. 1998; Cohen et al. 1999; Barrett et al. 2004; McCall et al. 2005; Koffarnus et al. 2011; Fernando et al. 2012; Watson et al. 2012).

### Data analysis

Alcohol intake and preference data for the initial 2 months of IAA were analyzed with two-way repeated-measures ANOVAs with week as the within-subject variable and group (LD;HD) as the between-subject variable. The effects of the pharmacological treatments were analyzed using three-way repeated-measures ANOVAs with time (2, 7 and 24 h) and dose as within-subject variables and group (LD;HD) as the between-subject variable. In case of a significant interaction effect involving the drug dose, follow-up two-way repeated-measures ANOVAs per time-point (2, 7 and 24 h) were conducted with dose as within-subject variable and group (LD;HD) as the between-subject variable. Post hoc pairwise comparisons of each drug dose with vehicle were performed with LSD tests. Mauchly’s test of sphericity was used to determine if variances of the differences between treatment levels were equal. If the assumption of sphericity was violated, degrees of freedom were corrected using Huynh-Feldt estimates of sphericity to more conservative values. Corrected degrees of freedom are presented rounded to the nearest integer. All statistical analyses were conducted using IBM SPSS Statistics for Windows, version 22.0 (IBM Corp., Armonk, NY, USA). The threshold for statistical significance was set at *p* < 0.05. All data are presented as mean ± SEM. Graphs were made using GraphPad Prism 6.

## Results

### Alcohol consumption during IAA in LD and HD

In agreement with our previous study (Spoelder et al. 2015), when comparing alcohol intake of the first month (7 h/day IAA) to the second month (24 h/day IAA), HD showed increased alcohol intake to a greater extent than to LD (batch 1: *F*_(1,30) month x group_ = 96.33, *p* < 0.001; batch 2: *F*_(1,10) month x group_ = 29.53, *p* < 0.001). Statistical analyses confirmed the group differences in alcohol intake and preference over the initial 2 months of IAA (batch 1: intake: *F*_(1,30) group_ = 179.78, *p* < 0.001; preference: *F*_(1,30) group_ = 208.34, *p* < 0.001; batch 2: intake: *F*_(1,10) group_ = 113.31, *p* < 0.001; preference: *F*_(1,10) group_ = 120.55, *p* < 0.001) (Table 1). Total fluid intake was not different between LD and HD (batch 1: *F*_(1,30) group_ = 0.39, n.s.; batch 2: *F*_(1,10) group_ = 3.34, n.s.) (data not shown).

**Table 1:** Alcohol intake and preference for HD and LD during the initial 2 months of IAA, prior to pharmacological treatment

		Alcohol intake		Alcohol preference	
		7 h/day	24 h/day	7 h/day	24 h/day
Batch 1	HD (*n* = 16)	2.61 ± 0.16	5.46 ± 0.25	46.84 ± 2.47	57.97 ± 2.40
	LD (*n* = 16)	1.00 ± 0.06	1.71 ± 0.12	17.11 ± 1.01	18.30 ± 1.42
Batch 2	HD (*n* = 6)	2.02 ± 0.15	5.25 ± 0.42	59.58 ± 3.87	60.04 ± 3.43
	LD (*n* = 6)	0.49 ± 0.08	1.30 ± 0.26	20.28 ± 4.91	17.30 ± 4.02

**Fig. 1:**
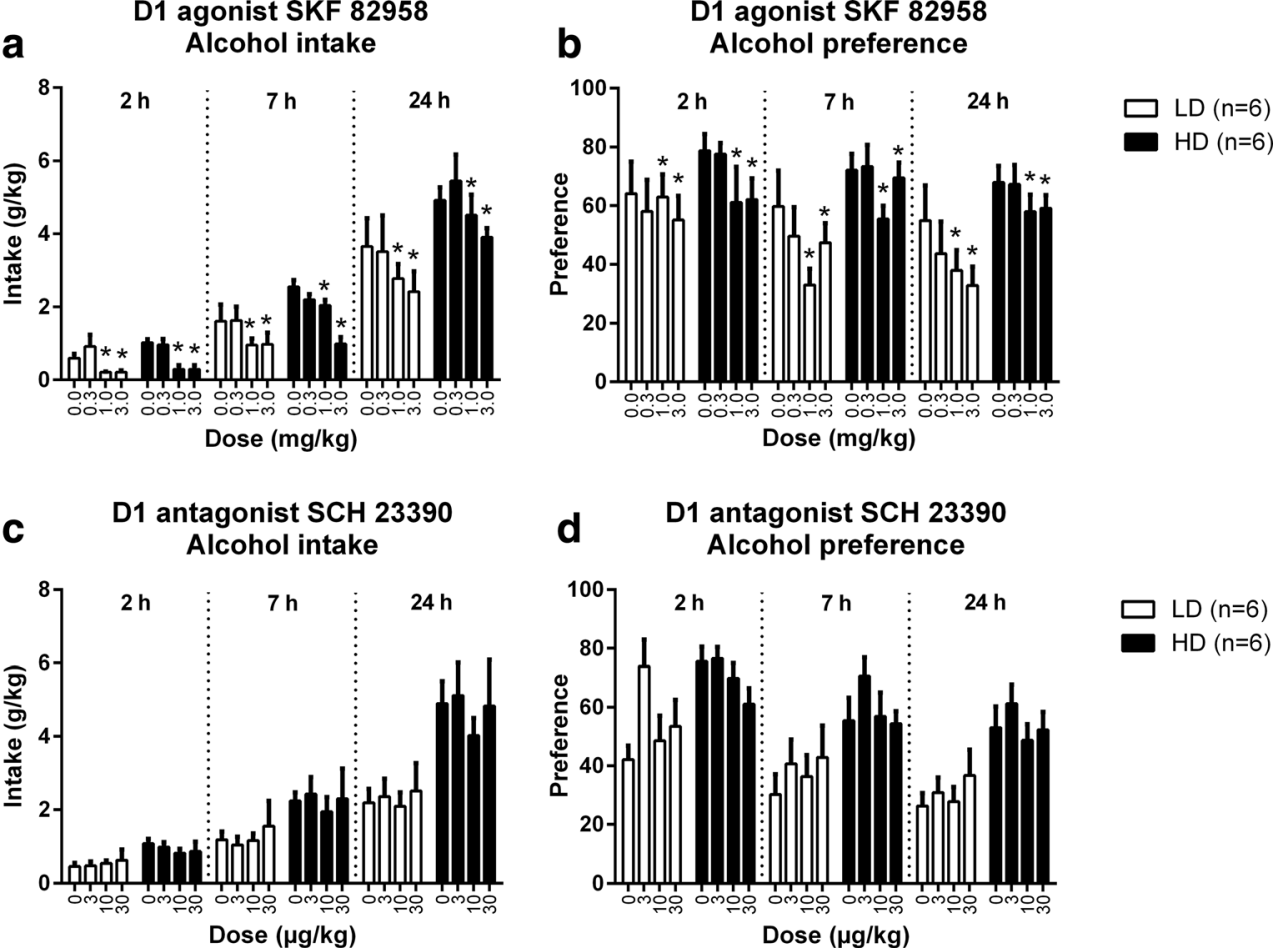
The effects of the dopamine D1 receptor agonist SKF 82958 and the dopamine D1 receptor antagonist SCH 23390 on alcohol intake and preference in HD and LD. SKF 82958 decreased alcohol intake and preference during the entire session to a similar extent in HD and LD (**a**, **b**). SCH 23390 did not alter alcohol intake (**c**). Alcohol preference was affected by SCH 23390 but post hoc analyses did not reveal significant differences from vehicle for any of the doses tested (**d**). HD consumed more alcohol than LD (with a near significant trend for SKF 82958): SKF 82958: *F*_(1,10) group_ = 4.83, *p* = 0.053, SKF 82958: *F*_(1,12) time x group_ = 4.88, *p* < 0.05; SCH 23390: *F*_(1,10) group_ = 16.09, *p* < 0.003, SCH 23390: *F*_(1,14) time x group_ = 17.62, *p* < 0.001. The preference for alcohol was also higher for HD compared to LD and was independent of session time (with a near significant trend for SKF 82958): SKF 82958: *F*_(1,10) group_ = 4.74, *p* = 0.055; SCH 23390: *F*_(1,10) group_ = 17.11, *p* < 0.003. Data are presented as the mean + SEM. The effect of SKF 82958 did not interact with the session time. Therefore, the *asterisk* reflects the overall differences from vehicle in post hoc pairwise comparisons (*p* < 0.05)

**Fig. 2:**
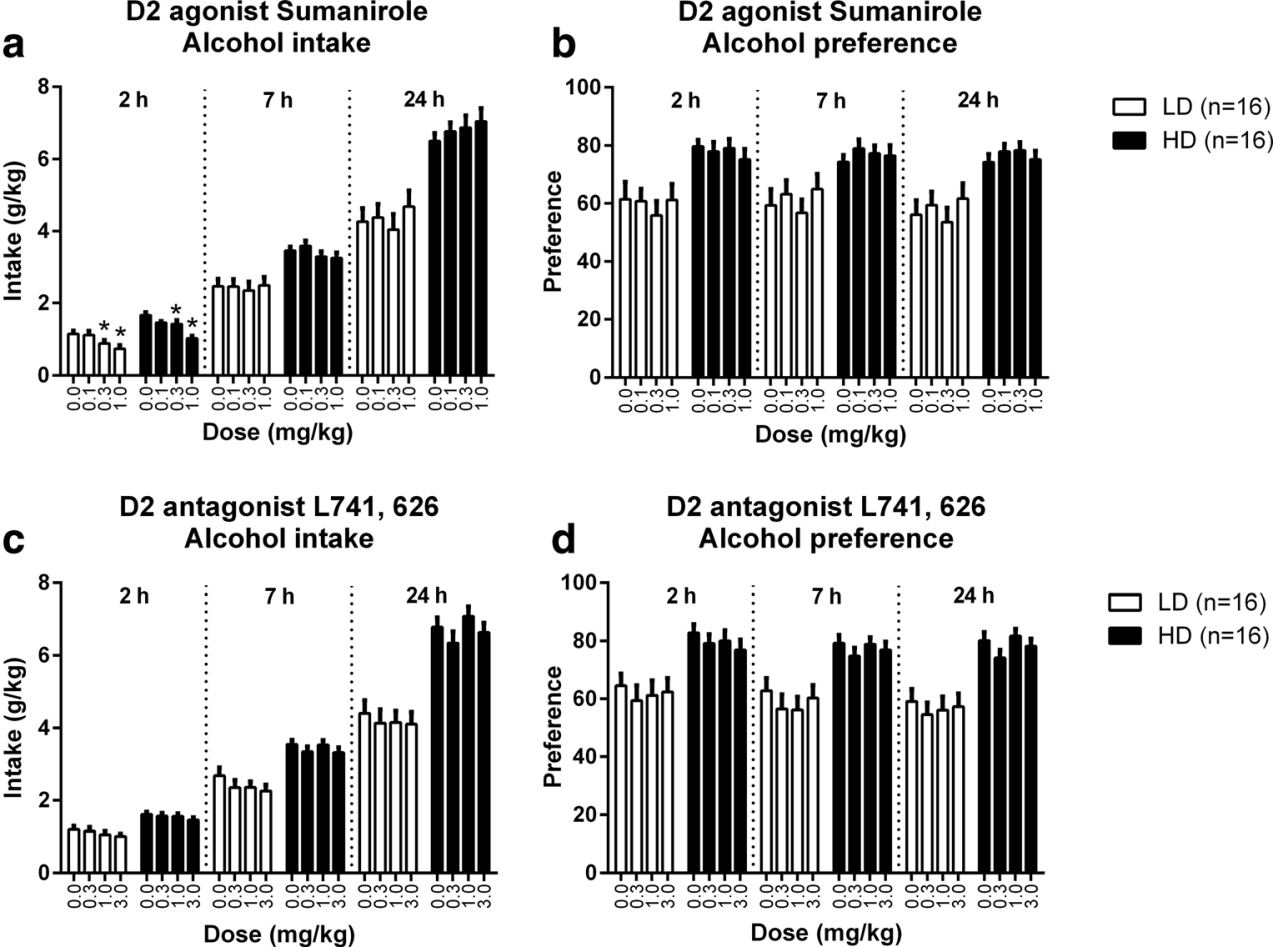
The effects of the dopamine D2 receptor agonist sumanirole and the dopamine D2 receptor antagonist L741,626 on alcohol intake and preference in HD and LD. Sumanirole decreased alcohol intake after 2 h of alcohol exposure in both groups, without affecting alcohol intake after 7 or 24 h of alcohol exposure (**a**). Sumanirole had no effect on the preference for alcohol (**b**). L741,626 did not affect alcohol intake and preference (**c**, **d**). HD consumed more alcohol than LD: Sumanirole: *F*_(1,30) group_ = 27.34, *p* < 0.001, *F*_(1,35) time x group_ = 29.78, *p* < 0.001; L741,626: *F*_(1,30) group_ = 38.51, *p* < 0.001, *F*_(1,37) time x group_ = 40.19, *p* < 0.001. The preference for alcohol was also higher for HD compared to LD and was independent of session time: Sumanirole: *F*_(1,29) group_ = 12.21, *p* < 0.003; *L*741,626: *F*_(1,30) group_ = 22.36, *p* < 0.001. Data are presented as the mean + SEM. *Asterisk* means different from vehicle in post hoc pairwise comparisons (*p* < 0.05)

**Fig. 3:**
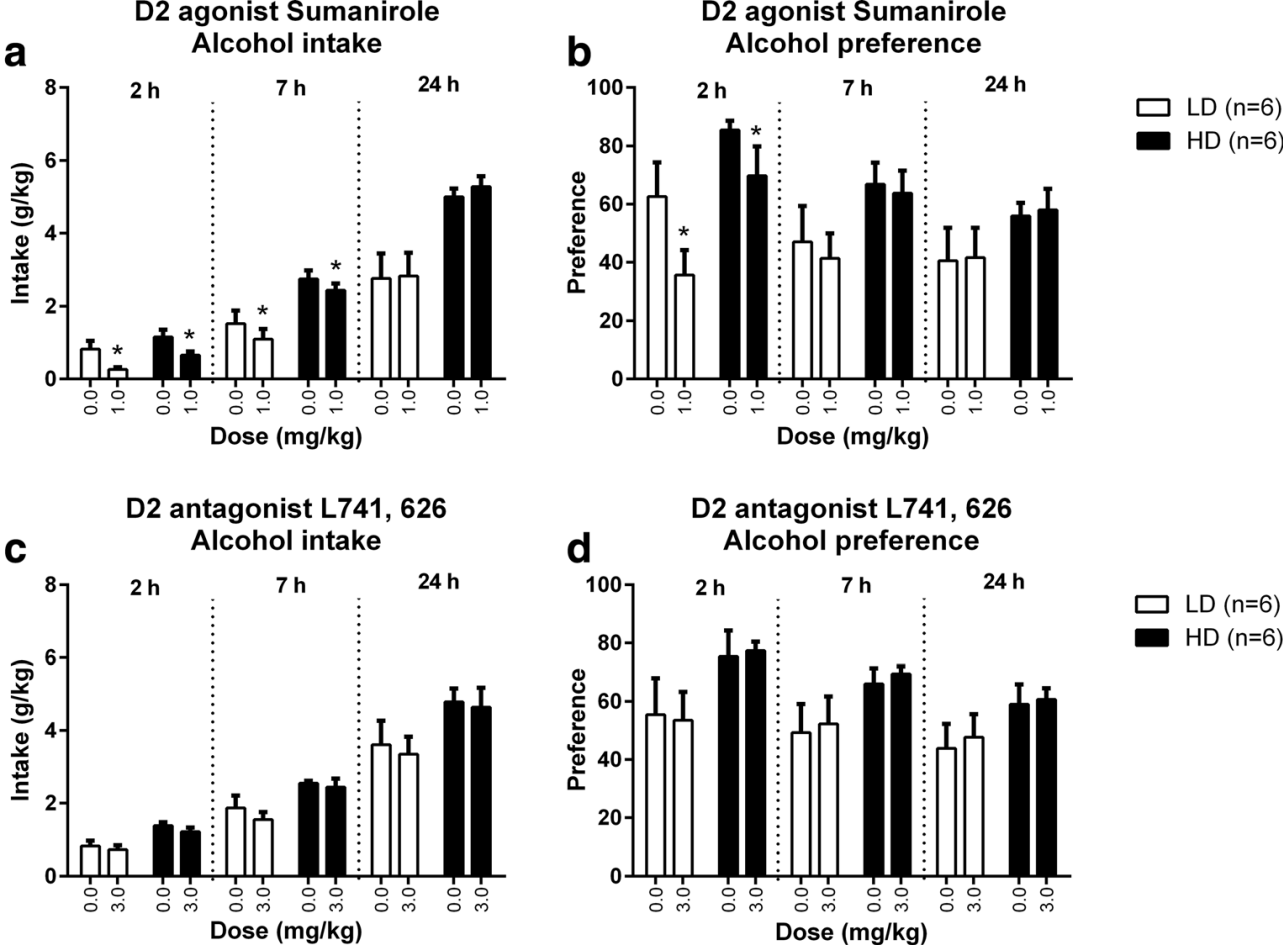
Replication of the effects of the highest dose of the dopamine D2 receptor agonist sumanirole and the dopamine D2 receptor antagonist L741,626 on alcohol intake and preference in HD and LD. Sumanirole decreased alcohol intake in both groups after 2 and 7 h of alcohol exposure, but was without effect after 24 h of alcohol exposure (**a**). Sumanirole decreased the preference for alcohol after 2 h of alcohol exposure but had no effects after 7 and 24 h of alcohol exposure (**b**). L741,626 did not affect alcohol intake and preference (**c**, **d**). HD consumed more alcohol than LD: Sumanirole: *F*_(1,10) group_ = 11.36, *p* < 0.008, *F*_(1,13) time x group_ = 11.80, *p* < 0.004; L741,626: *F*_(1,10) group_ = 5.82, *p* < 0.04, *F*_(1,13) time x group_ = 1.68, n.s. There were near significant trends for a higher preference for alcohol in HD compared to LD, and the preference was independent of session time: Sumanirole: *F*_(1,10) group_ = 4.03, *p* = 0.073; L741,626: *F*_(1,9) group_ = 4.51, *p* = 0.063. Data are presented as the mean + SEM. *Asterisk* means different from vehicle in post hoc pairwise comparisons (*p* < 0.05)

During the phase of treatment with the dopaminergic drugs, HD consumed more alcohol than LD (see figure legends). The differences in alcohol intake between HD and LD typically became more pronounced as the session progressed (significant time × group interaction for all compounds, except for L741,626 in the second batch). Preference for alcohol was also greater in HD than LD (significant effect of group for all compounds, with near significant trends for SKF 82958 and for the second batch treated with sumanirole and L741,626, independent of session time) (Figs. 1, 2 and 3).

### Dopamine D1 receptor agonist—SKF 82958

Treatment with SKF 82958 decreased alcohol intake (*F*_(3,30) dose_ = 9.58, *p* < 0.001), independent of session time (*F*_(6,55) dose x time_ = 1.43, n.s.) or group (*F*_(3,30) dose x group_ = 0.41, n.s.; *F*_(6,55) time x dose x group_ = 1.38, n.s.) (Fig. 1a). Post hoc analyses showed that alcohol intake was reduced after treatment with 1.0 and 3.0 mg/kg SKF 82958 (Fig. 1a).

SKF 82958 decreased the preference for alcohol (*F*_(3,30) dose_ = 4.04, *p* < 0.02), independent of session time (*F*_(5,49) dose x time_ = 1.33, n.s.) or group (*F*_(3,30) dose x group_ = 0.41, n.s.; *F*_(5,49) time x dose x group_ = 0.90, n.s.) (Fig. 1b). Post hoc analyses showed that the preference for alcohol was decreased after treatment with 1.0 and 3.0 mg/kg SKF 82958 (Fig.1b).

Because treatment with SKF 82958 reduced alcohol intake and preference after 24 h of alcohol exposure, we examined if SKF 82958 affected alcohol consumption in the subsequent re-baseline session, during which the animals received no treatment. Alcohol intake and preference during the re-baseline session were not affected by SKF 82958 treatment in the previous session (alcohol intake: *F*_(3,30) dose_ = 0.13, n.s.; preference: *F*_(3,30) dose_ = 0.20, n.s.) (data not shown).

### Dopamine D1 receptor antagonist—SCH 23390

Treatment with SCH 23390 did not affect alcohol intake (*F*_(3,30) dose_ = 0.27, n.s.) at any of the time points tested (*F*_(4,35) time x dose_ = 0.51, n.s.), independent of group (*F*_(3,30) dose x group_ = 0.20, n.s.; *F*_(4,35) time x dose x group_ = 0.14, n.s.) (Fig. 1c).

SCH 23390 treatment had no main effect on alcohol preference (*F*_(3,30) dose_ = 1.68, n.s.), but there was a three-way interaction with group and session time (*F*_(6,60) time x dose x group_ = 3.08, *p* < 0.02) (Fig. 1d). Subsequent analyses per time point indicated that SCH 23390 influenced the preference for alcohol during the first 2 h of the session (*F*_(3,30) dose 2 h_ = 2.99, *p* < 0.05), independent of group (*F*_(3,30) dose x group 2h_ = 2.04, n.s.), without a clear dose-dependent direction. Indeed, post hoc analyses did not reveal a significant difference of any of the doses of SCH 23390, when compared to vehicle. Alcohol preference was not affected by SCH 23390 after 7 h (*F*_(3,30) dose 7 h_ = 1.29, n.s.; *F*_(3,30) dose x group 7 h_ = 0.69, n.s.) and 24 h of alcohol exposure (*F*_(3,30) dose 24 h_ = 0.74, n.s.; *F*_(3,30) dose x group 24 h_ = 0.56, n.s.) (Fig. 1d).

### Dopamine D2 receptor agonist—Sumanirole

Treatment with sumanirole affected the level of alcohol intake, dependent on the time in the session (*F*_(5,136) time x dose_ = 9.29, *p* < 0.001), but independent of group (*F*_(5,136) time x dose x group_ = 1.55, n.s.) (Fig. 2a). Follow-up analyses per time point indicated that sumanirole decreased alcohol intake during the first 2 h of the session (*F*_(3,90) dose 2 h_ = 20.87, *p* < 0.001) to a similar extent in LD and HD (*F*_(3,90) dose x group 2 h_ = 1.68, n.s.). Post hoc analyses showed that alcohol intake was reduced after treatment with 0.3 and 1.0 mg/kg sumanirole (Fig. 2a). Alcohol intake was no longer affected by sumanirole after 7 h of alcohol access (*F*_(3,90) dose 7 h_ = 1.30, n.s.; *F*_(3,90) dose x group 7 h_ = 0.92, n.s.). By contrast, analyses of the entire 24 h showed a trend towards an increase in alcohol intake (*F*_(3,90) dose 24 h_ = 2.39, *p* = 0.074), independent of group (*F*_(3,90) dose x group 24 h_ = 0.95, n.s.) (Fig. 2a). Analysis of the alcohol consumption data between 2 and 24 h after session onset confirmed that alcohol intake was increased during the last 22 h of the session (*F*_(3,90) dose 2–24 h_ = 12.16, *p* < 0.001) in both groups (*F*_(3,90) dose x group 2–24 h_ = 0.99, n.s.) (data not shown).

The effects of sumanirole on alcohol intake were replicated in the second batch of animals (Fig. 3a, b), again revealing session time-dependent effects (*F*_(2,20) time x dose_ = 6.80, *p* < 0.007), independent of group (*F*_(2,20) time x dose x group_ = 0.07, n.s.). Subsequent analyses indicated that sumanirole decreased alcohol intake after 2 and 7 h (*F*_(1,10) dose 2 h_ = 13.03, *p* < 0.006; *F*_(1,10) dose 7 h_ = 7.38, *p* < 0.03) in both LD and HD (*F*_(1,10) dose x group 2 h_ = 0.05, n.s.; *F*_(1,10) dose x group 7 h_ = 0.21, n.s.), without affecting alcohol intake over the full 24 h of the session (*F*_(1,10) dose 24 h_ = 1.09, n.s.; *F*_(1,10) dose x group 24 h_ = 0.41, n.s.) (Fig 3a, b). Interestingly, alcohol intake increased between 2 and 24 h of exposure to alcohol (*F*_(1,10) dose 2–24 h_ = 10.96, *p* < 0.009) in both groups (*F*_(1,10) dose x group 2–24 h_ = 0.13, n.s.), similar to the results from the initial experiment (data not shown).

Sumanirole treatment did not affect alcohol preference in the first batch (*F*_(3,87) dose_ = 0.88, n.s.; *F*_(4,119) time x dose_ = 1.81, n.s.; *F*_(4,119) time x dose x group_ = 0.10, n.s.) (Fig. 2b), but did alter alcohol preference in the second batch (*F*_1,10) dose_ = 5.75, *p* < 0.04), independent of group (*F*_(1,10) dose x group_ = 0.53, n.s.). The effect of sumanirole on alcohol preference in the second batch was dependent on the time in the session (*F*_(1,15) time x dose_ = 9.53, *p* < 0.005), but was independent of group (*F*_(1,15) time x dose x group_ = 0.51, n.s.). Subsequent analyses for the second batch revealed that sumanirole decreased preference for alcohol after 2 h (*F*_(1,10) dose 2 h_ = 11.52, *p* < 0.008) but had no effects after 7 h (*F*_(1,10) dose 7 h_ = 1.21, n.s.) and 24 h of alcohol exposure (*F*_(1,10) dose 24 h_ = 0.30, n.s.), independent of group (2 h: *F*_(1,10) dose x group 2 h_ = 0.79, n.s; 7 h: *F*_(1,10) dose x group 7 h_ = 0.10, n.s; 24 h: *F*_(1,10) dose x group 24 h_ = 0.03, n.s.) (Fig. 3b).

### Dopamine D2 receptor antagonist—L741,626

There was a trend for an effect of L741,626 treatment on alcohol intake (*F*_(3,90) dose_ = 2.63, *p* = 0.055), independent of the time in the session (*F*_(4,124) time x dose_ = 1.85, n.s.) or the group (*F*_(4,124) time x dose x group_ = 1.04, n.s.) (Fig. 2c). L741,626 did not affect alcohol intake in the second batch (*F*_(1,10) dose_ = 1.38, n.s.; *F*_(1,15) time x dose_ = 0.05, n.s.; *F*_(1,15) time x dose x group_ = 0.13, n.s.) (Fig. 3c).

Treatment with L741,626 did not influence the rats’ preference for alcohol in the first (*F*_(3,90) dose_ = 1.58, n.s.; *F*_(5,141) time x dose_ = 0.56, n.s.; *F*_(5,141) time x dose x group_ = 0.52, n.s.) (Fig. 2d) or the second batch (*F*_(1,9) dose_ = 0.69, n.s.; *F*_(2,18) time x dose_ = 0.25, n.s.; *F*_(2,18) time x dose x group_ = 0.11, n.s.) (Fig. 3d).

## Discussion

In the present study, we found that treatment with the dopamine D1 receptor agonist SKF 82958 reduced alcohol intake and preference in rats. Treatment with the dopamine D2 receptor agonist sumanirole induced a transient reduction followed by an increase in alcohol intake. By contrast, the dopamine D1 and D2 receptor antagonists, SCH 23390 and L741,626, did not alter alcohol consumption. Interestingly, the effects of the dopamine D1 and D2 receptor agonists were similar in LD and HD, suggesting that individual variation in alcohol consumption does not involve altered dopamine signaling.

The reductions in voluntary alcohol consumption upon treatment with dopamine D1 and D2 receptor agonists are in agreement with previous studies (Linseman 1990; Dyr et al. 1993; George et al. 1995; Silvestre et al. 1996), despite differences in experimental procedures (e.g. continuous vs. intermittent alcohol access; sweetened vs. unsweetened alcohol, different alcohol concentrations, food restriction procedures, inclusion criteria, species and strain). Interestingly, the current study, as well as previous reports shows that dopamine D1 receptor agonists are more powerful in reducing alcohol intake than dopamine D2 receptor agonists (Linseman 1990; Ng and George 1994; Silvestre et al. 1996; El-Ghundi et al. 1998). After dopamine D1 receptor stimulation using SKF 82958, alcohol intake and preference was reduced throughout the session. In contrast, the selective dopamine D2 receptor agonist sumanirole mainly reduced alcohol intake during the first phase of the alcohol consumption session, and concurrently reduced preference for alcohol during the first 2 h of the session. Importantly, upon the initial decrement in alcohol intake, sumanirole increased alcohol intake during the remainder of the session. The initial decrease in alcohol intake, followed by a subsequent rise in alcohol intake after treatment with sumanirole, suggests a rebound effect after the initial suppression of alcohol intake. Importantly, however, a similar increment in alcohol intake did not occur upon SKF 82958 treatment, indicating that an initial decrease in alcohol intake is not necessarily followed by a rebound increase in alcohol intake. The behavioural effects of sumanirole have been reported to be longer in duration than those of SKF 82958 (Gnanalingham et al. 1995; McCall et al. 2005). Based on these kinetic profiles, a longer-lasting reduction in alcohol consumption upon sumanirole treatment would have been expected. Rather, we observed an initial decrement in alcohol consumption for both sumanirole and SKF82958, followed by an increase in alcohol intake for sumanirole. These effects are therefore unlikely to be explained by differences in the kinetics of the two compounds. Together, these data indicate that dopamine D1 and D2 receptors play different roles in the modulation of alcohol drinking, whereby dopamine D1 receptor stimulation evokes a clear-cut reduction in alcohol intake and preference.

Treatment with the dopamine D1 and D2 receptor antagonists SCH 23390 and L741,626 did not alter alcohol intake and preference. These findings are in agreement with the lack of effect of dopamine D1 and D2 receptor antagonists on voluntary alcohol consumption that has been reported previously (Brown et al. 1982; Goodwin et al. 1996; Silvestre et al. 1996). However, decreases in voluntary alcohol consumption upon treatment with either dopamine D1 and D2 receptor antagonists have been reported as well by several studies (Pfeffer and Samson 1986; Dyr et al. 1993; Panocka et al. 1995; El-Ghundi et al. 1998; Bulwa et al. 2011; Sabino et al. 2013), while only one study reported an increase in alcohol consumption (Dyr et al. 1993). Importantly, the doses that reduced alcohol consumption often also decreased water intake, possibly reflecting a non-specific suppression of fluid intake or a more general impairment in motor activity (Linseman 1990; Hubbell et al. 1991; Dyr et al. 1993). In any event, the lack of an effect of dopamine receptor antagonists on alcohol consumption suggests that endogenous dopamine does not play a primary role in alcohol consumption, at least not under IAA conditions.

Comparable dopamine receptor drug treatments have been performed in the context of operant alcohol self-administration. These studies show that treatment with dopamine D1 and D2 receptor agonists and antagonists reduced responding for alcohol, but not its actual consumption (Pfeffer and Samson 1988; Rassnick et al. 1993; Files et al. 1998; Cohen et al. 1999; Czachowski et al. 2001; Czachowski et al. 2002; Samson and Chappell 2004). Dopamine receptor agonists have been suggested to substitute for the reinforcing effects of alcohol (Hodge et al. 1993; Samson and Chappell 1999), whereas dopamine receptor antagonists may attenuate the reinforcing properties of alcohol (Imperato et al. 1987; Imperato and Di Chiara 1988; See et al. 1991; Santiago et al. 1993). Taken together with the consumption studies, these findings suggest that both dopamine D1 and D2 receptors are important for the regulation of alcohol intake when an effort is required to obtain alcohol (Salamone and Correa 2012).

Individual susceptibility to AUD has been related to dopamine receptor deficiency and an altered dopaminergic response to alcohol. Previous preclinical studies, for example, showed that alcohol-preferring rodents have reduced levels of dopamine in the terminal regions of the mesolimbic dopamine system (Murphy et al. 1987; Gongwer et al. 1989; McBride et al. 1990; George et al. 1995), which led to the hypothesis that their response to dopamine D1 or D2 receptor stimulation or inhibition might be altered. Interestingly, both humans at risk for AUD and rats bred or selected for high alcohol intake respond to alcohol exposure with greater increases in extracellular dopamine levels (Weiss et al. 1993; Katner and Weiss 2001; Doyon et al. 2005; Bustamante et al. 2008; Setiawan et al. 2014). However, in both AUD patients and social drinkers, treatment with a dopamine D2 receptor antagonist has been shown to reduce alcohol craving and to increase control over alcohol intake (Borg 1983; Modell et al. 1993; Peters and Faulds 1994; Enggasser and de Wit 2001; Martinotti et al. 2010). The effect of treatment with dopaminergic drugs on the subjective effects of alcohol has been shown to differ among individuals (Holdstock and de Wit 1998; Holdstock and de Wit 1999; Enggasser and de Wit 2001; Holdstock and de Wit 2001). For example, the dopamine D2 receptor antagonist haloperidol reduced the alcohol-induced euphoric effects in subjects who experienced stimulant effects upon alcohol intake, whereas these effects were absent in individuals who primarily reported sedative-like effects (Enggasser and de Wit 2001). In rodents, treatment with dopamine D1 and D2 receptor agonists and antagonists in alcohol-preferring animals resulted in similar changes in voluntary alcohol consumption as observed in outbred cohorts (Weiss et al. 1990; Dyr et al. 1993; George et al. 1995; Panocka et al. 1995; Goodwin et al. 1996; Sabino et al. 2013). The current findings are in line with these studies; the dopamine D1 and D2 receptor agonists and antagonists affected alcohol intake to a similar extent in LD and HD. Together, the current and previous findings suggest that individual differences in voluntary alcohol intake are not primarily related to alterations in dopaminergic signaling.

To conclude, treatment with both dopamine D1 and D2 receptor agonists reduced voluntary alcohol consumption, whereby the reduction in alcohol intake and preference was most pronounced after activation of dopamine D1 receptors. Thus, drugs that stimulate dopamine D1 receptors may aid in the treatment of AUD. Dopamine receptor antagonist treatment did not alter alcohol intake and alcohol preference, suggesting that endogenous dopamine is not essential for alcohol consumption under IAA conditions. Moreover, the comparable effects of dopamine D1 and D2 receptor agonists in LD and HD suggest that the individual level of alcohol intake is not related to differences in dopamine signaling. Taken together, these data increase our knowledge on the modulatory role of dopamine in alcohol intake.
